# Inhibition of RND-mediated efflux attenuates antibiotic resistance and virulence in hypervirulent *Klebsiella pneumoniae*

**DOI:** 10.1128/iai.00301-25

**Published:** 2025-09-22

**Authors:** Mia E. Van Allen, Yuding Weng, X. Renee Bina, James E. Bina

**Affiliations:** 1 Department of Microbiology and Molecular Genetics, University of Pittsburgh School of Medicine 12317 , Pittsburgh, Pennsylvania, USA; Stanford University School of Medicine, Stanford, California, USA

**Keywords:** *Klebsiella pneumoniae*, efflux, pathogenesis, antimicrobial resistance, capsule

## Abstract

*Klebsiella pneumoniae* (Kp) is a major human pathogen causing hospital-acquired and community-acquired infections with emerging hypervirulent strains (hvKp) posing a significant threat due to its ability to cause severe invasive infections in healthy individuals. In addition to antimicrobial resistance, virulence factors including capsule production, biofilm formation, and iron acquisition systems are critical for hvKp pathogenesis. In this study, we investigated how resistance-nodulation-division (RND)-family efflux systems contribute to antimicrobial resistance and virulence in hvKp strain KPPR1 using the RND-specific inhibitor phenyl-arginine β-naphthylamide (PAβN). We found that PAβN treatment rendered KPPR1 more susceptible to multiple antibiotics while simultaneously attenuating virulence factor production. PAβN significantly reduced capsule biosynthetic gene expression, resulting in decreased uronic acid levels, hypermucoviscosity, and biofilm formation. PAβN also impaired growth under iron-limited conditions, suggesting RND-mediated efflux contributes to iron acquisition. PAβN-dependent virulence attenuation was demonstrated through reduced KPPR1 adherence to cultured intestinal enterocytes and decreased pathogenicity in the *Galleria mellonella* infection model compared to untreated controls. Collectively, these results demonstrate that RND-mediated efflux is critical for both antimicrobial resistance and virulence in hvKp strain KPPR1. Our findings establish RND efflux inhibitors as promising dual-target therapeutics that can simultaneously combat antibiotic resistance and attenuate virulence in hvKp infections.

## INTRODUCTION

*Klebsiella pneumoniae* (Kp) is a gram-negative bacterium that is a major cause of healthcare-associated infections across the globe (1). Two distinct Kp pathotypes have emerged: classical Kp (cKp), which is multidrug-resistant (MDR) and primarily associated with nosocomial infections, and hypervirulent Kp (hvKp), which can cause community-acquired infections in healthy individuals. The virulence of hvKp strains is enhanced due to their acquisition of several virulence determinants, including regulators of hyper-capsule production, adhesins involved in biofilm formation, and genes encoding for multiple iron acquisition systems (1, 2). These virulence factors can exist chromosomally but are often identified in clinical isolates encoded within a mobilizable virulence plasmid (3, 4). The presence of these virulence factors collectively functions to facilitate immune evasion and persistence within the host, differentiating the pathogenic potential of hvKp strains from cKp strains. In contrast, cKp strains have become a global health threat due to their expression of diverse antibiotic resistance mechanisms, both intrinsic and acquired traits, that counteract antimicrobial agents (5). Alarmingly, the convergence of these two pathotypes through horizontal gene transfer has led to the emergence of MDR hypervirulent strains (5–7). These hybrid Kp strains represent a significant public health threat, underscoring the urgent need for novel therapeutic strategies.

The resistance-nodulation-division (RND) family of efflux pumps plays a crucial role in both antimicrobial resistance and virulence in gram-negative pathogens (8). These efflux systems are considered a primary mediator of intrinsic antimicrobial resistance in gram-negative bacteria (9). RND transporters form tripartite complexes composed of an inner membrane RND-family transporter, a periplasmic adaptor protein, and an outer membrane pore protein, homologous to TolC (10). Among RND transporters, many exhibit exceptionally broad substrate specificity, actively exporting multiple substrates that are chemically and structurally unrelated including antibiotics, bile salts, fatty acids, detergents, heavy metals, toxic metabolic byproducts, and quorum sensing signals (10, 11). By expelling toxic compounds, RND pumps enhance bacterial survival, allowing the cell to withstand diverse environmental stresses, including exposure to antibiotics and host-derived antimicrobial defenses (9). Additionally, RND-mediated efflux has been implicated in the regulation of virulence factors in multiple pathogens, including capsule biosynthesis, biofilm formation, iron homeostasis, and resistance to host immune clearance (12, 13). The extensive functional repertoire of RND transporters underscores their importance in bacterial adaptation, persistence, and pathogenesis, making them attractive targets for therapeutic intervention.

Kp, like most gram-negative bacteria, encodes multiple independent RND transporters. Analysis of the hypervirulent strain KPPR1 reveals 11 distinct RND efflux pump proteins organized into 10 operons (14). Notably, only one operon encodes a TolC homolog, suggesting the remaining transporters share TolC as their outer membrane pore protein (15). These RND transporters show significant sequence divergence, particularly in their substrate-binding pockets, which indicates that individual transporters may have distinct substrate specificities and unique physiological roles (13, 16, 17).

Two of these 11 transporters, AcrB and OqxB, have been studied for their contributions to antimicrobial resistance and virulence (12, 13, 18). Both transporters contribute to antimicrobial resistance with overlapping substrate profiles (13, 18). However, only AcrB enhances virulence in a murine pneumonia model (13). These findings demonstrate that while RND transporters can be functionally redundant for antimicrobial resistance, they make distinct contributions to both resistance and virulence. This diversity may result from differential expression in response to environmental conditions or, alternatively, differential contributions to the efflux of their respective native substrates (9). Functional redundancy such as this is common among RND efflux systems and complicates efforts to define the specific roles of individual Kp RND transporters and the relationship between the RND-mediated efflux and cell physiology (10).

An indirect method to assess RND efflux function on a systems level is to inactivate TolC. As TolC is required for the formation of active tripartite transporters, strains lacking *tolC* are effectively rendered RND negative (19). Previous studies using *tolC* mutants in both *Vibrio cholerae* and hvKp demonstrated antimicrobial hypersusceptibility phenotypes and virulence attenuation that was consistent with what is observed in RND-mediated efflux impaired cells (11, 20–22). In both *V. cholerae* and hvKp backgrounds, *tolC* mutation resulted in increased susceptibility to multiple antibiotics, impaired biofilm formation, virulence factor production, and attenuated virulence in infection models (11, 20). However, these results are not definitive, as TolC also serves as an essential outer membrane channel for other tripartite transporters, including the major facilitator superfamily (MFS) and ATP-binding cassette (ABC) transporters (15). Thus, the pleiotropic role of TolC complicates efforts to attribute specific phenotypes to RND-mediated efflux alone, necessitating alternative approaches using genetics or chemical genetics to interrogate the direct contribution of RND-mediated efflux to bacterial biology.

Given the important role of RND-mediated efflux in antimicrobial resistance, there has been significant interest in developing RND-specific chemical inhibitors (23, 24). These inhibitors could circumvent antimicrobial resistance by potentiating antibiotic activity in drug-resistant pathogens. Although several RND inhibitors have been described to date, none are approved for clinical or veterinary use (25). Nevertheless, these inhibitors represent important tools that facilitate chemical genetic approaches to interrogate RND contributions to antimicrobial resistance, cell physiology, and virulence. Understanding these relationships is particularly crucial given the emerging threat of MDR-hypervirulent Kp strains, which combine antibiotic resistance with enhanced pathogenicity, making infections exceedingly difficult to treat.

One such RND inhibitor is phenylalanine-arginine β-naphthylamide (PAβN) (26). PAβN functions as a competitive inhibitor of RND-mediated efflux by binding to the substrate-binding pocket of RND efflux pumps, thereby inhibiting substrate binding and subsequent efflux (27, 28). Its activity sensitizes bacteria to antibiotics (29, 30) and inhibits the efflux of native substrates of the RND transporters, affecting physiological processes dependent on efflux activity. Previous studies with PAβN in other gram-negative pathogens, such as *Pseudomonas aeruginosa*, *Acinetobacter baumannii*, and *V. cholerae*, have demonstrated its utility in defining RND efflux-specific contributions to antimicrobial resistance and pathogenesis (28, 29, 31, 32). Previous studies using PAβN in Kp have primarily focused on its potentiation of antimicrobials (30, 33, 34), highlighting the potential utility of PAβN in treating MDR isolates. However, these studies have not examined PAβN’s effects on virulence phenotypes. Given the critical role of RND-mediated efflux in the expression of virulence traits, we investigated the use of PAβN as a tool to dissect the contribution of RND-mediated efflux to hvKp antimicrobial resistance and pathogenesis.

Our results show that PAβN treatment significantly increases antibiotic susceptibility while impairing virulence-related phenotypes, including capsule production, biofilm formation, and iron acquisition. Consistent with these findings, PAβN treatment attenuated virulence in the *Galleria mellonella* infection model. These results indicate a central role for RND efflux in both resistance and pathogenesis and suggest that RND efflux inhibitors function as dual-target therapeutics that mitigate both antimicrobial resistance and virulence in Kp.

## RESULTS

### PAβN enhances antibiotic susceptibility

To define the contribution of RND-mediated efflux to Kp antimicrobial resistance, we assessed the effects of PAβN on the antimicrobial susceptibility of hvKp strain KPPR1Δ*bla* to a panel of antibiotics using the disk diffusion method. The Δ*bla* mutant, which contains an in-frame deletion of the chromosomal β-lactamase gene (35), was used as the wild-type (WT) strain in these studies to assess whether the RND efflux system contributes to resistance to β-lactam antibiotics. As TolC is required for RND transporter function, a previously characterized KPPR1 *tolC* mutant (*bla*+) was included as a putative RND-null control (20). Briefly, overnight cultures of the strains were spread onto the surface of Lysogeny Broth (LB) agar plates containing either 0 or 0.5 mM PAβN before specific antibiotic disks were placed onto the surface of the inoculated plates. The zones of growth inhibition for each antibiotic were then measured after overnight incubation at 37°C.

The results showed that the addition of PAβN significantly increased KPPR1 susceptibility to multiple antibiotics, as evidenced by increased zones of growth inhibition compared to untreated controls (Table 1). This included increased susceptibility to chloramphenicol, ciprofloxacin, erythromycin, norfloxacin, novobiocin, and trimethoprim. Similar susceptibility changes were observed with the *tolC* mutant, confirming that the increased antimicrobial susceptibility was not due to PAβN toxicity. Further support for this conclusion was provided by the observation that PAβN did not alter KPPR1 susceptibility to other antibiotics (e.g., colistin, imipenem, gentamicin, and polymyxin B; Table 1).

**TABLE 1:** Effect of PAβN treatment on antibiotic susceptibility of KPPR1

	Zone of inhibition in mm (mean ± SD)^*a*^		
Antibiotic	KPPR1S (WT)	0.5 mM PAβN	TolC mutant
Chloramphenicol	25.0 ± 0.0	**31.7 ± 0.6**	**32.3 ± 0.6**
Ciprofloxacin	24.7 ± 0.6	**29.3 ± 0.6**	**31.7 ± 0.6**
Colistin	12.0 ± 0.0	13.3 ± 0.6	12.0 ± 0.0
Erythromycin	6.0 ± 0.0	**30.7 ± 0.6**	**18.7 ± 0.6**
Gentamicin	19.0 ± 1.0	20.0 ± 0.0	18.7 ± 0.6
Imipenem	27.7 ± 0.6	29.7 ± 0.6	27.0 ± 1.0
Norfloxacin	22.7 ± 0.6	**26.0 ± 1.0**	**29.3 ± 0.6**
Novobiocin	11.3 ± 1.2	**21.3 ± 1.2**	**20.3 ± 0.6**
Polymyxin B	12.7 ± 0.6	14.7 ± 0.6	13.0 ± 0.0
Tigecycline	19.3 ± 0.6	**24.3 ± 0.6**	**22.0 ± 0.0**
Trimethoprim	18.3 ± 0.6	**29.7 ± 0.6**	**27.7 ± 0.6**

^*a*^ Values represent the mean ± standard deviation (SD) from three independent experiments. Statistical significance was assessed using one-way analysis of variance with Dunnett’s *post hoc* test to compare each group to the WT control. Bold values denote statistically significant differences from WT (*P* < 0.05).

Erythromycin and novobiocin demonstrated the most dramatic changes in their resistance profiles. Both PAβN treatment and *tolC* deletion resulted in substantial increases in susceptibility compared to the KPPR1 control, consistent with previous studies that identified RND-mediated efflux as a major contributor to erythromycin and novobiocin resistance in other members of the Enterobacteriaceae (36–38). Interestingly, the marked difference in the erythromycin zone of growth inhibition between the *tolC* mutant (18.7 ± 0.6 mm) and PAβN-treated cells (30.7 ± 0.6 mm) suggests that *tolC* deletion may trigger compensatory adaptations that contribute to erythromycin resistance (19, 30, 39).

Based on these results, we concluded that chloramphenicol, ciprofloxacin, erythromycin, norfloxacin, novobiocin, and trimethoprim were substrates of the Kp RND systems, and that the RND systems did not contribute to colistin, gentamicin, imipenem, and polymyxin B resistance.

### RND efflux inhibition reduces capsule production, hypermucoviscosity, and biofilm formation

Capsule production, hypermucoviscosity (HMV), and biofilm formation are critical Kp virulence determinants (1). Previous studies demonstrated that mutation of *tolC* in KPPR1 attenuated these virulence phenotypes (20). However, the contribution of RND-mediated efflux to the aforementioned phenotypes is not known. To address this gap, we cultured KPPR1 in the presence of PAβN and quantified capsule production, HMV, and biofilm formation. We hypothesized that if RND-mediated efflux contributed to capsule polysaccharide (CPS) production, selective inhibition by PAβN would attenuate the CPS-related phenotypes, similar to that which was observed in the *tolC* mutant.

**Fig 1:**
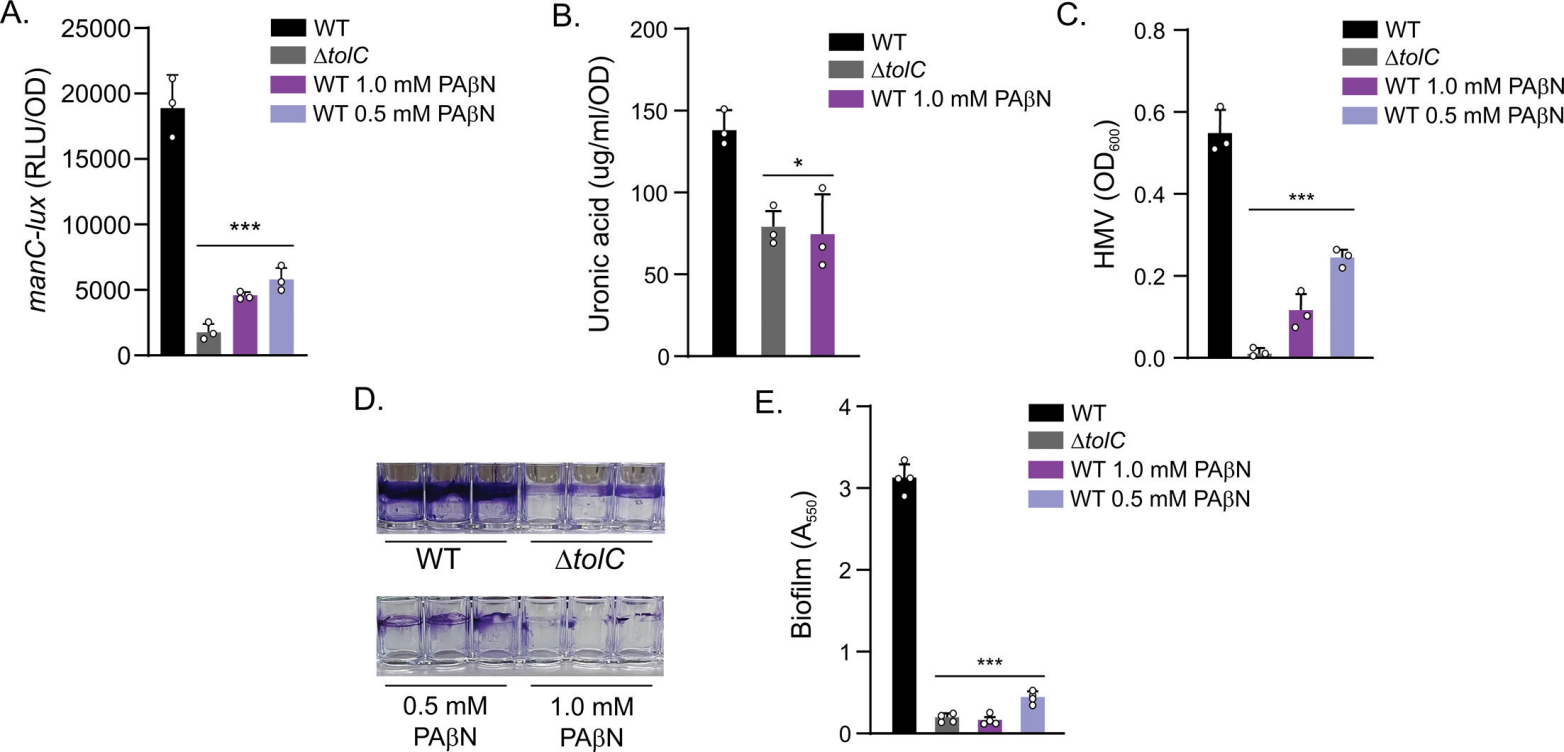
RND efflux inhibition attenuates capsule production, hypermucoviscosity (HMV), and biofilm formation in *K. pneumoniae*. (**A**) Growth in the presence of PAβN decreases expression of the capsule biosynthetic gene *manC* at various time points compared to untreated KPPR1. The KPPR1 *tolC* mutant shows a more pronounced reduction in *manC* expression compared to PAβN-treated cells. (**B**) Uronic acid levels were measured to quantify capsule production. Growth in PAβN and deletion of *tolC* both result in significantly reduced uronic acid concentration. No significant difference was observed between 0.5 mM and 1.0 mM PAβN treatments. (**C**) Treatment with PAβN results in reduced HMV phenotype similar to *tolC* deletion, with a concentration-dependent effect as measured by the sedimentation assay. (**D**) Representative photograph of crystal violet-stained biofilms formed by wild-type KPPR1, the *tolC* mutant, and wild type treated with 0.5 and 1.0 mM PAβN. (**E**) Quantification of biofilm formation from D following destaining with 30% acetic acid and measuring absorbance at 550 nm. All results represent the mean and standard deviation of at least three independent biological replicates. The biofilm staining results in D are representative of three experiments. Data for A, B, C, and E represent the mean and standard deviation of three independent replicates. Statistical significance compared to WT was determined by two-tailed unpaired *t*-test (A) or unpaired *t*-test with Welch’s correction (B, C, and E). *P < 0.05, ***P > 0.001.

To test this, we first examined the expression of a key capsule biosynthesis gene, *manC*, using luminescence-based transcriptional reporters (40). The *manC* promoter is frequently used as a reporter for the expression of the Kp CPS operon (41). PAβN treatment significantly inhibited *manC* expression compared to WT, with a 3.25-fold reduction at 0.5 mM and a 4.11-fold reduction at 1.0 mM (Fig. 1A). This indicated that RND-mediated efflux is required for high-level expression of the CPS biosynthetic genes. By contrast, the *tolC* mutant showed the most pronounced reduction in expression compared to both WT (10.75-fold) and PAβN-treated conditions. This finding confirms the pleiotropic effects of TolC and suggests that additional TolC-dependent tripartite transporters (i.e., ABC or MF transporters) likely also contribute to CPS gene expression.

We next quantified capsule production by measuring uronic acid, a major carbohydrate component of the KPPR1 capsule (Fig. 1B) (42, 43). WT KPPR1 produced the highest uronic acid concentration (~340 µg/mL/OD_600_). Treatment with PAβN significantly reduced uronic acid production to ~225 µg/mL/OD_600_, which was comparable to levels observed in the *tolC* mutant (~250 µg/mL/OD_600_). These results provide additional evidence that RND-mediated efflux is necessary for high-level capsule production and bolster the *manC* expression results described above.

HMV is an important virulence trait in hvKp (41, 42). hvKp strains typically exhibit a hyper-mucoid phenotype that plays a critical role in pathogenesis that is distinct from capsule production (42–44). HMV can be quantified using a sedimentation assay, where the sedimentation rate of individual clones is inversely proportional to the level of HMV, with high HMV strains remaining in the culture supernatant following low-speed centrifugation relative to low HMV strains that pellet more efficiently (42). This difference can then be measured by determining the OD_600_ of the culture supernatant following low-speed centrifugation. Using the sedimentation assay to measure HMV (Fig. 1C), we observed a PAβN concentration-dependent reduction in HMV relative to wild type. By contrast, the *tolC* mutant showed the most dramatic decrease in HMV, confirming a previous report (20). Given that PAβN-treated cells showed an intermediate phenotype—with decreased HMV compared to wild type but greater than the *tolC* knockout mutant—these results suggest that while RND efflux contributes to HMV, it is not the sole determinant of this phenotype, and that other TolC-dependent transporters also contribute to HMV.

Biofilm formation is critical for Kp pathogenesis (45). We evaluated the effect of PAβN on KPPR1 biofilm production using crystal violet staining (Fig. 1D and E). WT KPPR1 produced robust biofilms, while PAβN-treated cells and the Δ*tolC* mutant showed significantly reduced biofilm formation, both visually (Fig. 1D) and quantitatively (Fig. 1E). The PAβN-treated and Δ*tolC* mutant biofilms retained less crystal violet dye (OD_550_ of ~0.200–0.450) compared to WT (OD_550_ of ~3.00). Treatment with 1.0 mM PAβN resulted in decreased biomass compared to treatment with 0.5 mM PAβN (OD_550_ of 0.200 vs 0.450), with the higher concentration producing an effect like that of the Δ*tolC* mutant (OD_550_ of ~0.200 vs 0.210). These results suggest that RND-mediated efflux contributes to biofilm formation.

### RND efflux inhibition impairs growth under iron-limiting conditions

Iron acquisition is a critical virulence determinant in hvKp. Hypervirulent strains produce 3–4 different siderophores, compared to cKp counterparts which produce 1–2 different siderophores (1). Given previous reports demonstrating the contribution of RND-mediated efflux to iron homeostasis and siderophore secretion (21, 46–48), we investigated whether PAβN affected iron homeostasis in KPPR1 by quantifying the effect of PAβN on KPPR1 growth under iron-limiting conditions. We hypothesized that if RND systems were involved in siderophore secretion, their inhibition by PAβN would compromise siderophore secretion and result in growth attenuation under iron-limiting conditions.

**Fig 2:**
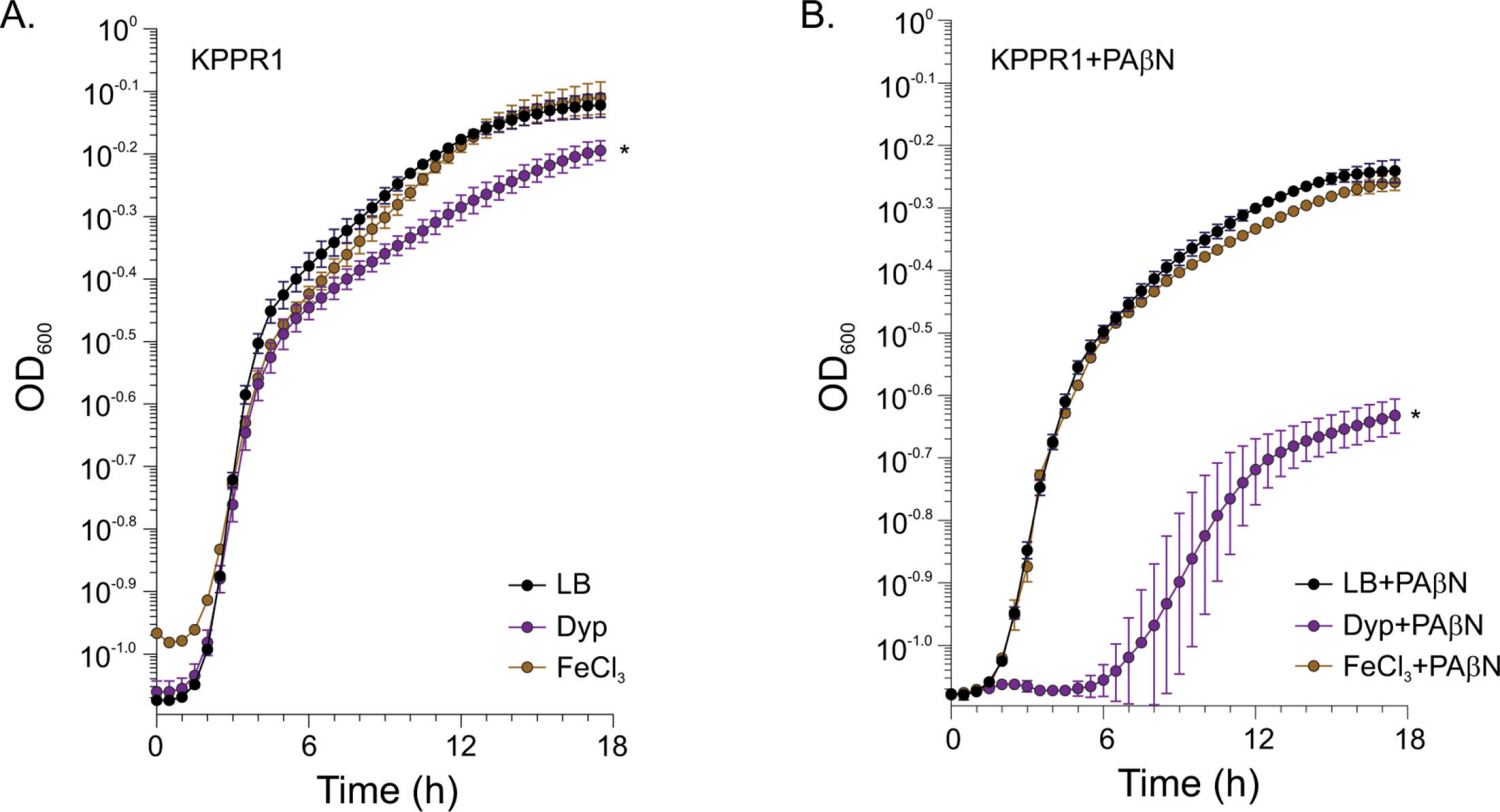
PAβN attenuates growth in iron-limited conditions. WT KPPR1 was cultured in three different media conditions: standard LB medium, iron-depleted LB medium (containing 130 µM 2,2′-dipyridyl), and iron-replete LB medium (containing 130 µM 2,2′-dipyridyl plus 130 µM FeCl_3_). Cultures were grown (**A**) with PAβN or (**B**) without PAβN (0.5 mM or 1.0 mM) at 37°C with shaking in a microtiter plate. Optical density at 600 nm was measured every 30 minutes for 18 hours. The iron-replete condition served as a control to demonstrate that growth inhibition was due to iron limitation rather than dipyridyl toxicity. Data represent the mean ± standard deviation of three independent replicates. The area under the curve (AUC) was determined for each growth curve and compared to the untreated control under the same experimental condition using an unpaired *t*-test with Welch’s correction. *P < 0.05 compared to the untreated control.

To test this, we cultured WT KPPR1 in LB medium, iron-depleted LB (130 µM 2,2′-dipyridyl), and iron-replete LB (130 µM 2,2′-dipyridyl plus 130 µM FeCl_3_), with and without PAβN. We monitored growth over time as the change in OD_600_. The results showed that KPPR1 exhibited reduced growth in iron-depleted conditions compared to LB medium (Fig. 2A). Adding equimolar iron restored growth to LB-equivalent levels, confirming that the growth effects were due to iron limitation rather than dipyridyl toxicity. Adding PAβN to iron-depleted medium significantly impaired growth, extending the lag phase to 6 hours and reaching a final OD_600_ of 0.23, compared to WT which had a lag phase of ~2 hours and reached a final OD_600_ of 0.57 (Fig. 2B). Supplementation with equimolar FeCl_3_ restored growth kinetics to WT levels, indicating that the growth impairment was due to iron limitation, rather than PAβN or 2,2′-dipyridyl toxicity.

Collectively, these results demonstrate that PAβN inhibition of RND efflux impairs the cells' ability to acquire iron, resulting in reduced growth in iron-limited media and suggesting a specific role for RND efflux systems in iron metabolism, likely through their role in siderophore secretion.

### PAβN attenuates virulence in *G. mellonella* and affects attachment to enterocytes

To evaluate whether the observed *in vitro* attenuation of virulence factors described above translated to reduced virulence *in vivo*, we assessed KPPR1 virulence using the *G. mellonella* infection model. This invertebrate model has been extensively validated with Kp and demonstrates concordance with mammalian infection outcomes (49, 50)

**Fig 3:**
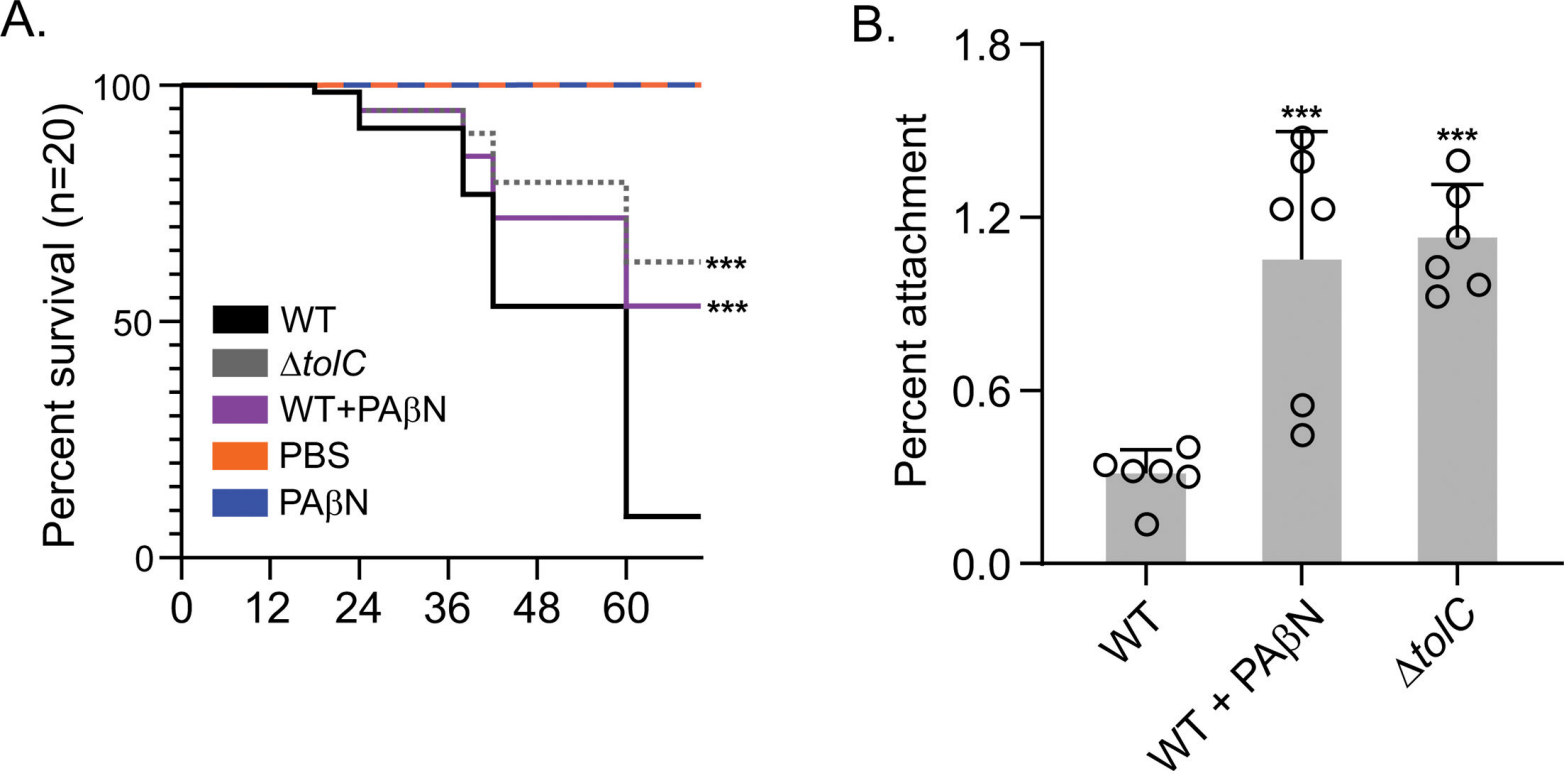
PAβN attenuates KPPR1 virulence and promotes epithelial cell adherence. (**A**) Infection of *G. mellonella* wax worm larvae with PAβN-treated KPPR1 results in significantly increased host survival compared to untreated controls (*P* < 0.001). The *tolC* mutant demonstrates similar virulence attenuation. Injection of sterile 1.0 mM PAβN in water does not induce larval death, confirming the inhibitor is not toxic to the host at this concentration. Statistical significance relative to the WT was determined using a log-rank (Mantel-Cox) test in GraphPad Prism version 9.4.1. ***P < 0.005. (**B**) KPPR1 attachment to HT29-MTX-E12 enterocytes. Both PAβN-treated KPPR1 and the *tolC* mutant show increased adherence to HT29-MTX-E12 cells compared to untreated wild-type cells. The data are the means and standard deviations of three independent experiments. Statistical significance was determined using a one-way analysis of variance with Dunnett’s test, comparing each mean value to that of the WT. ***P < 0.005.

The results of these studies showed that larvae infected with PAβN-treated KPPR1 showed significantly improved survival over 5 days compared to those infected with untreated bacteria (Fig. 3A). While approximately 80% of larvae infected with untreated KPPR1 succumbed to infection within 72 hours, 50% of larvae receiving PAβN-treated bacteria survived the entire experimental period. Notably, the survival rates of larvae infected with PAβN-treated KPPR1 closely matched those infected with the *tolC* mutant, suggesting that chemical inhibition of RND efflux systems is as effective as genetic disruption in attenuating virulence in this model. Injection of PAβN alone did not alter larval survival, confirming that the inhibitor at this concentration was not detrimental to the host.

We hypothesized that the PAβN-dependent inhibition of virulence factor production documented above likely contributed to attenuated virulence and increased larval survival. Since previous studies have indicated that capsule production contributes to immune evasion by inhibiting phagocytosis and clearance (51, 52), we tested whether the reduced capsule production observed in PAβN-treated KPPR1 affected bacterial attachment to an epithelial cell line.

For these assays, we used cultured human colon adenocarcinoma cells of the mucus-secreting HT29-MTX subclone, a well-characterized model for studying bacterial-enterocyte interactions due to their production of a thick adherent mucus layer (51, 53). As shown in Fig. 3B, the attachment of PAβN-treated KPPR1 cells to HT29-MTX cells was significantly higher than that of untreated wild-type cells (1% vs 0.3% adherence, respectively). This phenotype was similar to what was observed with the *tolC* mutant, which also showed approximately 1% adherence compared to 0.3% for the WT.

## DISCUSSION

In this study, we provide compelling evidence that RND-mediated efflux is critical for both antimicrobial resistance and virulence in hvKp. Our findings demonstrate that chemical inhibition of RND efflux by PAβN simultaneously compromises multiple Kp phenotypes, extending beyond antibiotic resistance to include virulence factors that are essential for host adaptation and immune evasion. This mechanistic link between RND-mediated efflux and pathogenesis has important implications for developing novel therapeutic approaches against hvKp and other gram-negative pathogens.

The convergence of antimicrobial resistance and hypervirulence in Kp represents a critical health challenge that necessitates novel treatment strategies. We found that PAβN treatment inhibited RND-mediated efflux and sensitized KPPR1 to multiple antibiotics, effectively phenocopying an hvKp *tolC* mutant (20). Our results are consistent with previous studies demonstrating that PAβN enhances antimicrobial susceptibility in Kp (30, 33, 34). For example, Hasdemir et al. reported that PAβN restored susceptibility to quinolones, chloramphenicol, and tetracycline in cKp (30). Vera-Leiva et al. evaluated PAβN effects in carbapenemase-producing Kp strains and found that, while PAβN lowered MICs for ciprofloxacin, it unexpectedly increased carbapenem resistance in multiple isolates, a phenotype they attributed to altered porin production (33). This contrasts with our results, in which PAβN did not increase resistance to any antibiotics tested but consistently potentiated their activity. The divergence among these results may reflect genetic variation among the strains, including the presence and absence of acquired resistance determinants in the isolates examined. The study by Pacios et al. focused on the combination of the anthelmintic drug niclosamide and PAβN, demonstrating the efficacy of PAβN to potentiate the activity of this drug, which is not used for treating bacterial infections, against Kp (34). Their findings highlight the potential utility of RND inhibitors in repurposing drugs to treat MDR infections. Collectively, the present results in combination with these studies provide compelling evidence that RND efflux inhibitors have the potential to restore antibiotic susceptibility in Kp by targeting RND-mediated efflux. Importantly, our work further establishes a major role for RND efflux in virulence (see below), highlighting efflux inhibition as a potential dual therapeutic strategy that may simultaneously inhibit both resistance and pathogenicity.

The contributions of RND-mediated efflux extended beyond antimicrobial resistance to attenuate virulence (13, 16, 39, 54). Our results demonstrated that RND efflux inhibition reduces expression of *manC*, a key gene within the cps regulon that serves as a reporter for the expression of capsule biosynthesis genes. This reduced expression likely explains the observed decrease in uronic acid content and overall capsule production in PAβN-treated cells. Similarly, the *tolC* mutant showed decreased *manC* expression, consistent with previous findings that TolC influences capsule locus expression (20). These findings suggest that RND-mediated efflux is required for robust expression of the capsule biosynthetic operons.

Interestingly, while both capsule and HMV were reduced by RND efflux inhibition, they appear to be regulated through partially distinct mechanisms. While the reduction in capsule production likely contributes to diminished HMV, it is important to note that *rmpC* and *rmpD* have been shown to regulate HMV development without affecting capsule production (41, 55). This suggests that RND efflux may influence these phenotypes through both shared and independent regulatory pathways. Additional studies are needed to determine this.

Biofilm formation, which is influenced by both capsule and HMV (45, 56, 57), was also significantly reduced by PAβN treatment. This aligns with our findings in the *tolC* mutant (20) and with previous work showing that efflux pumps contribute to biofilm development in Kp and other gram-negative bacteria (58). The interdependence of these phenotypes suggests that RND inhibition affects a common regulatory pathway affecting all three virulence traits.

The exact mechanism by which RND-mediated efflux affects these CPS-related phenotypes remains to be elucidated. One potential mechanism involves impaired efflux leading to altered intracellular concentrations of secondary messenger molecules or feedback inhibition due to accumulated metabolites normally exported by RND systems. Previous findings that RND transporters efflux autoinducers (59, 60) and cell metabolites (61, 62) are consistent with this hypothesis. Additional studies are warranted to address this topic, as it could reveal potential new avenues for therapeutic intervention in MDR strains.

RND efflux systems contribute to pathogenesis in some organisms through their function in iron homeostasis (21), and our findings suggest that this function is conserved in Kp. Iron is an essential nutrient for bacterial pathogens, and hvKp strains encode multiple siderophore systems to compete for iron within the host environment (1, 2). KPPR1 produces three siderophores: enterobactin, salmochelin, and yersiniabactin (1). Our results demonstrate that PAβN-treated cells were significantly attenuated for growth in low iron conditions, suggesting that functional RND efflux is required for proper iron acquisition. This finding aligns with previous studies in the Enterobacteriaceae that have shown RND efflux pumps contribute to siderophore secretion, with AcrB contributing to enterobactin, salmochelin, and yersiniabactin export (46, 47). Similarly, in *V. cholerae*, the VexH RND efflux pump exports vibriobactin, and disruption of this system results in altered cellular homeostasis (21) comparable to what we observed with PAβN-treated KPPR1. The *tolC* mutant was also attenuated for growth in low iron, consistent with TolC’s documented role in siderophore secretion (63).

We hypothesize that PAβN inhibition of RND efflux impairs iron homeostasis by directly inhibiting RND-mediated siderophore secretion. While the individual contributions of specific Kp RND efflux systems to iron homeostasis remain uncharacterized, KPPR1 encodes 11 RND pump proteins, including three independent AcrB homologs (14). Given the role of AcrB in siderophore secretion in other members of the Enterobacteriaceae (46, 47, 63), one or more of these homologs likely contribute to siderophore export in Kp. Future studies targeting individual RND systems in Kp will be important to determine their specific contributions to iron acquisition and homeostasis.

Having established that RND efflux inhibition attenuates multiple virulence factors *in vitro*, we assessed the *in vivo* relevance of our finding in the *G. mellonella* infection model. PAβN-treated KPPR1 showed significantly reduced virulence, leading to increased larval survival. This attenuation closely mirrored the phenotype of the *tolC* mutant, supporting our conclusion that RND-mediated efflux is essential for full virulence in hvKp. This conclusion is bolstered by previous work in a murine pneumonia model, where an *acrB*-deficient strain showed impaired lung colonization (12, 13). Together, these results provide strong evidence that RND efflux is an important virulence determinant in hvKp and suggests that RND efflux inhibitors represent potential antivirulence therapeutics.

Interestingly, we observed an inverse relationship between virulence and adherence to enterocytes. Using HT29-MTX cells to model gastrointestinal colonization, we found that PAβN-treated bacteria exhibited increased cell adherence, similar to the *tolC* mutant (20). This is consistent with reduced capsule production, as the capsule can mask adhesins in hvKp (52). When capsule synthesis is impaired, as seen with RND inhibition, adhesins become exposed, leading to enhanced adherence to epithelial cells (51, 64). However, this exposure also compromises immune evasion, as capsule-deficient bacteria are more readily recognized and cleared by the host immune system (51, 52, 64, 65). We hypothesize that while RND inhibition increases bacterial adherence, the concurrent reduction in capsule production and iron acquisition capabilities renders the bacteria more susceptible to immune clearance. This likely explains the decreased virulence observed in the *G. mellonella* model.

The results of this study raise new questions about the mechanism linking RND-mediated efflux to the adaptive responses involved in virulence gene regulation. While further investigations are needed, our findings align with a recently proposed model in *V. cholerae* where RND-mediated efflux functions primarily in the export of cellular metabolites (54, 66). When RND efflux is impaired, these metabolites accumulate intracellularly and activate environmental sensors, including one-component and two-component regulatory systems, triggering adaptive responses and transcriptional remodeling (16, 21, 22, 54). This disruption of cellular homeostasis appears to have broad consequences for pathogenic traits, including capsule production, iron acquisition, and immune evasion. This model may be broadly applicable across gram-negative pathogens, highlighting the therapeutic potential of RND efflux inhibitors as dual-function agents that both potentiate antibiotic activity and suppress virulence. This model helps explain why targeting RND efflux produces effects that extend well beyond antibiotic susceptibility. Importantly, this model appears to be broadly applicable across gram-negative pathogens where RND-mediated efflux has been linked to adaptive responses involved in pathogenesis (39, 48, 54), suggesting that RND efflux pump inhibitors represent promising dual-target therapeutics that may prove particularly valuable in combating emerging hypervirulent and multidrug-resistant pathogens like hvKp.

## MATERIALS AND METHODS

### Bacterial strain and culture conditions

The hypervirulent *Klebsiella pneumoniae* strain KPPR1 (14) and its isogenic *tolC* mutant were used for all experiments. The Δ*tolC* mutant, whose phenotypes for antimicrobial resistance, capsule production, biofilm formation, and virulence have been previously characterized and complemented (20), is included here as a control to confirm RND efflux dependence and to assess PAβN specificity. Cultures were grown in Luria-Bertani (LB) broth or on LB agar plates at 37°C unless otherwise specified. For capsule quantification and biofilm assays, bacteria were cultured statically in LB medium supplemented with PAβN (Alfa Aesar) at final concentrations of 0.5 mM or 1.0 mM, as indicated. The PAβN concentrations used were selected based on our determination that the PAβN MIC for KPPR1 was 1,000 mg/L, which is consistent with prior studies demonstrating PAβN MICs of 500–1,000 mg/L in multiple *K. pneumoniae* clinical isolates (34). PAβN was dissolved in water and used at the specified concentrations unless otherwise stated. Iron-limited conditions were established by supplementing LB with 200 µM 2,2′-dipyridyl, with or without 100 µM ferric chloride.

### Antimicrobial sensitivity testing

The antimicrobial susceptibility of *K. pneumoniae* KPPR1S was assessed using the disk diffusion method. Bacterial cultures were adjusted to a 0.5 McFarland standard and evenly spread onto Mueller-Hinton agar plates, with or without PAβN as indicated. Antibiotic-impregnated disks (BD) were placed on the agar surface, and plates were incubated at 37°C for 18–24 hours. Zones of inhibition were measured using a rule to 0.1 mm to determine antimicrobial susceptibility. A *tolC* mutant strain was included as a control for comparative analysis.

### Capsule assay

Capsular polysaccharides were extracted following modified protocols from Mike et al. and Walker et al. (41, 43, 55). Briefly, overnight bacterial cultures (0.5 mL) were mixed with 100 µL of 1% Zwittergent 3-14 detergent in citric acid buffer (100 mM, pH 2.0) and incubated at 50°C for 20 minutes. After centrifugation (16,000 × *g*, 5 minutes), 0.3 mL of clarified supernatant was combined with 1.2 mL of absolute ethanol. The mixture was incubated on ice for 20 minutes to precipitate capsular polysaccharides, followed by centrifugation (16,000 × *g*, 5 minutes). Precipitated polysaccharides were then resuspended in 0.2 mL of deionized water.

Uronic acid was quantified using the modified carbazole assay. Each sample (0.2 mL) was mixed with 1.2 mL sodium tetraborate solution (12.5 mM in concentrated H_2_SO_4_) and heated at 100°C for 5 minutes. After cooling to room temperature, 0.7 mL of each sample was combined with either 10 µL of 3-hydroxydiphenyl reagent (0.15% in 0.5% NaOH) or 10 µL of 0.5% NaOH (reagent blank). Absorbance was measured spectrophotometrically at 520 nm. Uronic acid concentrations were determined using a standard curve (0–100 μg/mL glucuronic acid; Sigma-Aldrich) and normalized to culture density (OD_600_). A previously characterized KPPR1 *tolC* mutant served as a positive control for reduced capsule production, while untreated KPPR1 cultures provided baseline capsule measurements (20).

### Biofilm assay

Biofilm formation was quantified using a crystal violet retention assay as previously described (20). Briefly, overnight bacterial cultures were diluted 1:100 in fresh LB broth, supplemented with or without PAβN, and 200 µL aliquots were transferred to sterile 96-well polystyrene microtiter plates. Plates were incubated statically at 37°C for 24 hours to allow biofilm development. Following incubation, planktonic cells were removed by gentle aspiration, and wells were washed three times with 220 µL sterile water (room temperature) to remove non-adherent bacteria. Plates were air-dried for 1 hour at room temperature before staining with 220 µL of 1% crystal violet solution for 10 minutes. After removing excess stain by three successive water washes, bound crystal violet was solubilized in 200 µL of 30% acetic acid. Biofilm formation was quantified by measuring absorbance at 590 nm using a microplate spectrophotometer. All experiments included technical triplicates and were performed in at least three independent biological replicates. Sterile LB broth served as a background control, and a previously characterized *tolC* mutant strain was included as a positive control for reduced biofilm formation (20).

### Sedimentation assay

Bacterial cultures were grown on an LB plate at 37°C for 18 hours. Individual colonies were selected in triplicate and grown overnight in LB broth at 37°C for 18 hours. Broth cultures were normalized by OD_600_, and 1 mL of normalized culture was centrifuged at 1,500 × *g* for 5 minutes. Care was taken not to disturb pellet formation when 600 µL of supernatant was taken from each culture, and OD_600_ was measured.

### Transcriptional reporter assays

For promoter activity measurements, overnight cultures of reporter strains bearing *manC-lux* or the empty vector were diluted 1:1,000 in fresh LB medium supplemented with chloramphenicol (20 µg/mL) to maintain plasmid selection (40). Aliquots (200 µL) were transferred to black-walled, clear-bottom 96-well microtiter plates and incubated at 37°C with continuous shaking. Luminescence (relative light units, RLU) and bacterial growth (OD_600_) were measured at regular intervals using a BioTek Synergy 4 microplate reader. Expression data were normalized to cell density (RLU/OD_600_), and each condition was tested in technical triplicates across three independent biological replicates. Empty vector controls were included to account for background luminescence.

### Iron-limitation growth analysis

The role of RND efflux in iron acquisition was evaluated using iron chelation and supplementation assays. KPPR1 and an isogenic *tolC* mutant were grown overnight in iron-limited LB medium containing 130 µM 2,2′-dipyridyl (DIP). For iron repletion controls, cultures were supplemented with 130 µM FeCl_3_. Overnight cultures were diluted 1:1,000 into fresh medium, maintaining the same iron conditions. To assess efflux inhibition effects, KPPR1 cultures were treated with PAβN at 1 mM or 0.5 mM. Growth kinetics were monitored in 96-well microtiter plates containing 200 µL culture per well. Optical density (OD_630_) measurements were recorded at 30 minute intervals for 20 hours using a BioTek microplate reader with continuous orbital shaking (200 rpm) at 37°C. All conditions were tested in technical triplicates across three independent biological replicates.

### *Galleria mellonella* infection model

Virulence was assessed using a *G. mellonella* larval infection model. Larvae (250–300 mg) were randomly assigned to experimental groups (*n* = 20 per group). Bacterial inoculums were prepared from overnight cultures of KPPR1 grown in LB broth with or without 1 mM PAβN. Cultures were washed twice with sterile phosphate-buffered saline (PBS) and adjusted to 10^6^ CFU/mL. Inoculum titers were verified by plating serial dilutions. Larvae were injected with 10 µL bacterial suspension (10⁴ CFU/larva) into the last right proleg using a Hamilton syringe. Following injection, larvae were maintained in sterile petri dishes with food at 25°C in darkness. Survival was monitored every 24 hours for 5 days. Mortality was defined as the absence of movement in response to tactile stimulation and the development of melanization. Control groups included (i) PBS-injected larvae, (ii) PAβN-only (500 µg/mL) treated larvae, (iii) unmanipulated larvae, and (iv) larvae infected with a previously characterized *tolC* mutant. Survival data were analyzed using Kaplan-Meier curves and log-rank tests for statistical comparison between groups.

### Enterocyte attachment assay

KPPR1 and its isogenic Δ*tolC* mutant strains were cultured overnight in LB at 37°C. The next day, 1 mL of culture from each strain was centrifuged, and the bacterial pellets were washed twice with Hank’s balanced salt solution (HBSS). Washed bacteria were then resuspended in 1 mL of HBSS buffer. HT29-MTX-E12 cells were cultured in 12-well plates for 3 weeks to achieve confluent monolayers. The monolayers were washed three times with HBSS, and 1 mL of Dulbecco’s modified Eagle medium (DMEM) was added to each well. For the PAβN treatment group, PAβN was added to a final concentration of 0.5 mM. Three empty wells without cultured cells received DMEM alone and were used to calculate the initial bacterial input. A 10 µL aliquot of the washed and resuspended bacteria was added to each well. All plates were incubated at 37°C for 2 hours. After incubation, wells containing HT29-MTX-E12 cells were washed five times with HBSS to remove non-adherent bacteria. The cells were then scraped in 1 mL of HBSS and collected. The resulting cell suspensions were serially diluted in HBSS, and dilutions were spread onto LB agar plates. The plates were incubated at 37°C overnight, and colonies were counted to determine the number of CFUs. Bacterial attachment was calculated as the ratio of adherent bacteria (CFU recovered from cell-containing wells) to the total bacterial input (CFU recovered from cell-free control wells).

### Statistical analysis

All experiments were conducted in biological triplicates, and data were analyzed using GraphPad Prism software. Statistical significance was assessed using unpaired Student’s *t*-tests for pairwise comparisons and one-way analysis of variance for multiple-group comparisons, followed by *post hoc* testing where applicable. A *P* value <0.05 was considered statistically significant. Data are presented as mean ± standard deviation unless otherwise specified.
